# Functional Characterization of *β*-Glucuronidase Genes Involved in Baicalein Biosynthesis from *Scutellaria baicalensis* Based on Transcriptome Analysis

**DOI:** 10.3390/ijms26051793

**Published:** 2025-02-20

**Authors:** Xin Zuo, Ping Li, Guangxi Ren, Zhenfang Bai, Dan Jiang, Chunsheng Liu

**Affiliations:** School of Chinese Materia Medica, Beijing University of Chinese Medicine, Beijing 102488, China

**Keywords:** *Scutellaria baicalensis*, *β*-glucuronidases (GUS), baicalein

## Abstract

Baicalein is a unique flavonoid compound with important pharmacological activities, derived from *Scutellaria baicalensis* Georgi. Baicalein, as the aglycone of baicalin, is a key form for exerting pharmacological activity in vivo. *β*-glucuronidases (GUSs) are the enzymes involved in the conversion of baicalin to baicalein. In this study, the content of baicalein in *S. baicalensis* was significantly increased by 20.44% after treatment with 5% PEG6000. Seven *GUSs* from the glycoside hydrolase 79 family were identified through comparative transcriptome analysis. Among them, GUS1 and GUS2 were confirmed to have catalytic activity in converting baicalin to baicalein in prokaryotic and eukaryotic systems. The correlation analysis further revealed a significant positive correlation of 0.962 (*p* < 0.01) between the expression of *GUS2* and baicalein content in six different sources of *S. baicalensis*. Interestingly, the presence of variable sites in the *GUS1* and *GUS2* genes significantly affected their catalytic efficiency in the *S. baicalensis* samples from the six geographic origins. These findings also provide valuable GUS biological enzyme resources for the effective synthesis of baicalein and offer new insights into the accumulation pattern of baicalein in *S. baicalensis*.

## 1. Introduction

*Scutellaria baicalensis* Georgi belongs to the genus of *Scutellaria* in the Labiatae family, and its root is a traditional Chinese herbal medicine commonly used in clinical practice [1]. Flavonoids are the primary active compounds of *S. baicalensis*, known for their diverse biological activities, including effective anti-inflammatory [2], anti-cancer [3], and antiviral [4] properties. Among them, baicalein is an active compound that exerts therapeutic effects in vivo or in clinical applications, such as the suppression of cancer cells [5], antioxidation [6], and resistance to depression [7]. In addition, baicalein is also regarded as a prospective anti-tumor [8] and antiviral drug [9], with important developmental and applicational value in clinical treatment. Therefore, baicalein is often used as an active pharmaceutical ingredient in various dosage forms in clinical applications, including tablets, oral liquids, injections, and granules.

However, the supply of baicalein is currently insufficient, necessitating the development of new methods to obtain it efficiently. *β*-Glucuronidase (GUS) is an important hydrolytic enzyme that plays a crucial role in the hydrolysis of glucuronide compounds [10]. Among them, endogenous GUS from the glycoside hydrolase 79 family plays an important role in the biosynthesis of baicalein in *S. baicalensis* [11,12]. Ikegami reported that GUS activity in *S. baicalensis* was optimal at pH 4.7 [13], while Sasaki identified the full-length sequence of the *GUS* gene from the hairy root of *S. baicalensis* with an open reading frame (ORF) of 1581 bp [12]. Huang further purified and functionally characterized the glucuronidase from *S. baicalensis* [14]. These studies suggest that the *GUS* gene is directly involved in baicalein synthesis and accumulation. The content of baicalein in *S. baicalensis* is closely related to the activity of both UBGAT (UDP-glucuronosyltransferase) and GUS. GUS facilitates the initial conversion of baicalin to baicalein under stress conditions, while UBGAT helps regulate the flavonoid glycoside balance by converting baicalein and its derivatives back to glycosylated forms. This mechanism helps prevent excessive reactive oxygen species and maintains normal physiological conditions [15]. Despite this, the baicalin content in *S. baicalensis* significantly exceeds that of baicalein, indicating that UBGAT is highly efficient in converting baicalein back to baicalin [16]. This suggests that the role of UBGAT in determining baicalein content is minimal, whereas GUS activity is likely the critical factor influencing baicalein levels. To date, there remains a gap in understanding the specific association between GUS activity and active compound content in *S. baicalensis*. Further research into this relationship is necessary to elucidate the regulatory mechanisms underlying baicalein biosynthesis and to develop strategies for enhancing its production.

Plant defense systems are activated when exposed to biotic or abiotic stresses, such as exogenous hormones, UV light exposure, pests and diseases, and mechanical damage. These stressors alter the activity of enzymes within the secondary metabolic network, leading to the accumulation of various secondary metabolites that help protect the plant [17,18]. Drought stress, a common abiotic stress, triggers the production of reactive oxygen species (ROS) in plants, which can cause oxidative cell damage. To counteract this, plants synthesize flavonoids, which play a critical role in mitigating oxidative damage [19,20]. In *S. baicalensis*, drought conditions have been shown to increase flavonoid concentrations significantly [21,22]. The relative content of major flavonoids in *S. baicalensis* exhibits a trend of first increasing and then decreasing as drought stress intensifies. Similarly, the activity of key enzymes involved in flavonoid biosynthesis follows this pattern, peaking under moderate drought stress before declining under more severe conditions [23,24]. Altogether, appropriate levels of drought stress can effectively enhance gene expression and promote the accumulation of secondary metabolites in plants.

*Escherichia coli* has been extensively used as a cellular host for expressing foreign proteins due to its rapid growth rate and low production costs [25]. Numerous commercial expression systems are available, designed to meet different experimental needs and ensure compatibility [26]. Prokaryotic expression systems, such as those based on *E. coli*, are widely utilized for large-scale protein expression, protein purification, and functional analyses in vitro [27,28,29]. Beyond the advantages of *E. coli*, such as its ease of culture, rapid growth, and cost-effectiveness, yeast expression systems offer additional benefits. The enriched inner membrane system in yeast allows for the secretion of some proteins into the extracellular environment. This capability enables the production of recombinant proteins that are not only soluble and appropriately folded but that also undergo essential post-translational modifications necessary for their proper function [26]. The methylotrophic yeast *Pichia pastoris* has emerged as an excellent eukaryotic expression host and is widely used for the recombinant production of both prokaryotic and eukaryotic proteins [30]. Constructing secondary metabolic synthetic pathways in yeast has proven to be a powerful tool in synthetic biology research. For instance, bioactive compounds, such as liquiritin, have been successfully synthesized in yeast [31,32]. Employing yeast expression systems for studying gene function provides a robust theoretical foundation for advancing synthetic biology and developing innovative metabolic engineering strategies.

This study first treated *S. baicalensis* seedlings with 5% PEG6000 and measured their baicalein content. Further, endogenous *GUS* genes that may be involved in baicalein biosynthesis were screened by transcriptome sequencing, and the functions of the target genes were characterized by prokaryotic and eukaryotic expression. This study provides a theoretical foundation for analyzing the biosynthesis of baicalein in *S. baicalensis* and provides biological enzyme resources for further effective acquisition of baicalein.

## 2. Results

### 2.1. Comparative Transcriptome Analysis of S. baicalensis After Stress Treatment

**Figure 1:**
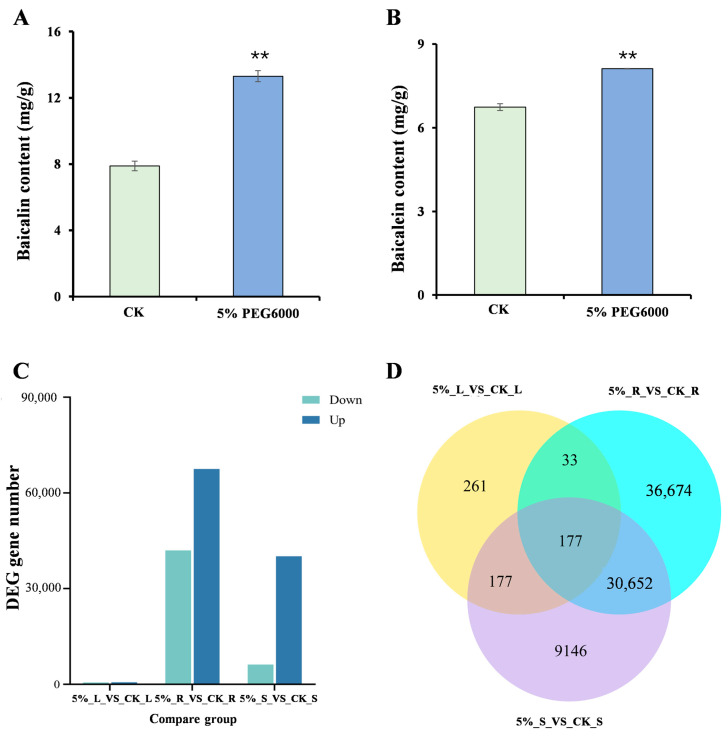
Effective ingredient content and differentially expressed genes in *S. baicalensis* treated with 5% PEG6000. (**A**) The content of baicalin in *S. baicalensis* roots treated with 5% PEG6000. (**B**) The content of baicalein in *S. baicalensis* roots treated with 5% PEG6000. (**C**) Differential gene numbers in different tissues of *S. baicalensis* roots (R), stems (S), and leaves (L) treated with CK and 5% PEG6000. (**D**) Venn diagram analysis of differentially expressed genes. The overlapping part of the graph represents the common differentially expressed genes between different comparison groups. CK: H_2_O-treated. “**” indicates *p* < 0.01.

The effective components in *S. baicalensis* roots treated with 5% Macrogol 6000 (PEG6000) were analyzed. The results show that the content of baicalin and baicalein in the *S. baicalensis* roots significantly (*p* < 0.01) increased after treatment with 5% PEG6000, with an increase of 68.72% and 20.44% compared to the control group (Figure 1A,B).

The leaves, stems, and roots of *S. baicalensis* were further compared by transcriptome analysis between the control group (H_2_O) and the treatment group (5% PEG6000). The transcriptome results demonstrate high-quality sequencing and reliable data for functional analyses. The clean reads ratio across all samples ranged from 94% to 97%, with an error rate of 0.02% to 0.03%. The GC content varied between 43.96% and 47.99%. Quality scores were also high, with Q20 ranging from 97.89% to 98.33% and Q30 from 93.85% to 94.89% (Table S1). As shown in Table S2, a total of 312,177 transcripts were generated for the two treated samples, with a mean length of 1034 bp. The N50 length was 1712 bp, and the N90 was 404 bp. Additionally, 186,042 unigenes were identified, with a mean length of 745 bp. The N50 and N90 lengths for the unigenes were 926 bp and 353 bp, respectively (Table S2). These metrics indicate that the transcriptome sequencing and assembly were robust, providing a solid basis for downstream analyses.

An analysis of differentially expressed genes (DEGs) across the different groups revealed that PEG6000 stress had the most significant impact on root gene expression (Figure 1C,D). In roots, the number of up-regulated genes was 1.6 times higher than that of down-regulated genes. Drought stress also notably affected gene expression in stems, with 46,348 DEGs, but had a minimal impact on leaves, where 40,152 DEGs were observed.

The unigenes were annotated to the Non-Redundant Protein Sequence Database (NR), Nucleotide Transcript Annotation (NT), Gene Ontology (GO), EuKaryotic Orthologous Groups (KOG), the Kyoto Encyclopedia of Genes and Genomes (KEGG), Swissprot, and Pfam, respectively (Figure S1A). Among the annotated unigenes, the NR, GO, Swissprot, and Pfam databases had higher annotation proportions compared to the others. Approximately 76.82% of the unigenes were annotated in at least one database, with an overall annotation proportion of 76.83%, leaving 23.17% of the unigenes unannotated. The GO annotation results (Figure S1B) indicate that in biological processes, the largest number of unigenes were involved in cellular processes, followed by metabolic processes. For cellular components, the cellular anatomical entity had the highest unigene count. In the molecular function category, the largest number of unigenes were associated with binding, followed by catalytic activity. This result indicates that the transcriptome data obtained in this study contained more genes related to metabolism and catalytic activity. The KOG annotation results (Figure S1C) indicate that the largest unigenes were annotated with translation, ribosomal structures, and biogenesis, suggesting enhanced physiological activities, such as protein synthesis, under stress conditions. The physiological characteristics of the samples used in this study were consistent with those expected after drought stress. A KEGG pathway analysis revealed that translation was the most prominent functional category. Furthermore, 885 unigenes were identified as being involved in secondary metabolic biosynthesis pathways (Figure S1D), highlighting the significance of secondary metabolism under drought stress.

### 2.2. Bioinformatics Analysis of GUSs

The gel electrophoresis results of PCR show that seven candidate *GUS* genes (*GUS1*–*GUS7*) were successfully cloned from *S. baicalensis* (Figure S2). The bioinformatics analysis results show that the ORF length of these genes ranged from 1065 bp to 1614 bp, respectively (Table S3). The predicted molecular weights of the GUS proteins ranged from 39.15 kDa to 59.30 kDa. The outcomes of the conserved domain prediction indicate that all seven candidate GUS proteins belong to the glycosyl hydrolase 79 superfamily, suggesting their involvement in enzymatic processes related to glycosyl hydrolysis (Table S4). The predicted properties of the GUS proteins (GUS1–GUS7) revealed that they were hydrophilic proteins and structurally stable (Table S4). The secondary structure prediction (Figure S3A–G) revealed that these proteins consist of four primary secondary structures, of which -helix (h), extended chain (e), -fold (t), and irregularly coiled coil (c) were the fundamental structural elements. According to the predicted structure of the 7 GUS proteins, GUS1–GUS7 had similar 3D structures that closely matched hyaluronoglucuronidase (SMTL ID: 7eyo.1), indicating that these proteins may have similar functions.

### 2.3. Phylogenetic Analysis of GUSs

**Figure 2:**
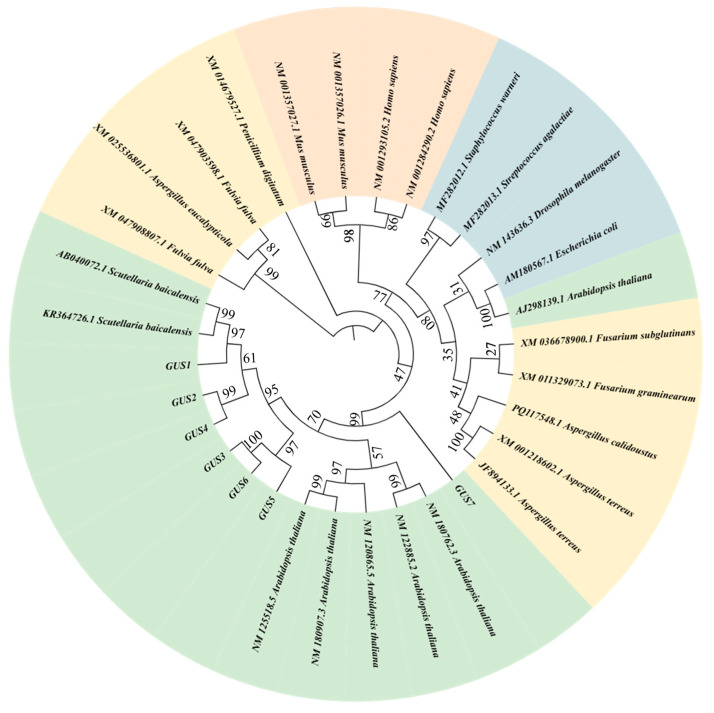
Phylogenetic analysis of the amino acid sequences of *GUSs* from different organisms.

Homologous sequences of *GUS* genes were searched for on NCBI, and an N-J tree was constructed by MEGA 10 to further analyze the evolutionary relationships of *GUSs*. The research results indicate that *GUSs* were categorized into four groups based on their different sources of fungi (yellow), bacteria (blue), animals (pink), and plants (green) (Figure 2). Among them, the seven cloned *GUSs* formed one cluster with plant-derived *GUSs*. Further analysis revealed that GUS1, GUS2, and GUS4 have relatively high similarities with previously reported endogenous GUS (AB040072 and KR364726) proteins in *S. baicalensis*, which belong to the GH79 family and have the function of hydrolyzing baicalin to baicalein. GUS5, GUS3, and GUS6 were clustered together and share a 97% similarity with each other. However, GUS7 has a distant phylogenetic relationship with six other GUS proteins from *S. baicalensis*. These results further demonstrate the conservation of GUS proteins in different species. In addition, GUS1, GUS2, and GUS4 may have potential catalytic activity for the hydrolysis of baicalin.

### 2.4. Characterization of Recombinant Proteins and Catalytic Activity

**Figure 3:**
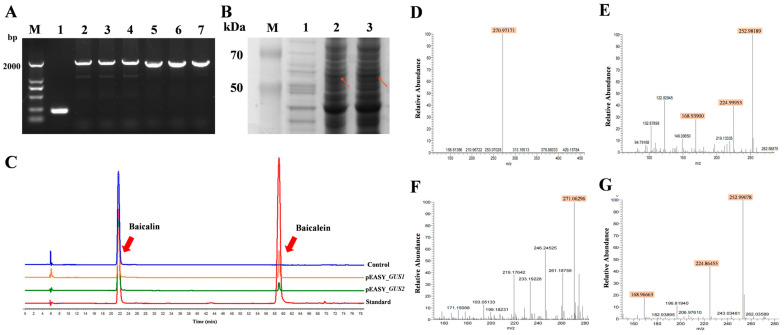
Results of prokaryotic expression experiments. (**A**) PCR electrophoresis results of ligated prokaryotic expression vectors (M: Marker; 1: BL21 (DE3)/E1; 2–4: BL21(DE3)/E1-*GUS1*; 5–7: BL21 (DE3)/E1-*GUS2*). (**B**) The results of SDS-PAGE for prokaryotic expression (M: Marker; 1: BL21 (DE3)/E1; 2: BL21 (DE3)/E1-*GUS1*; 3: BL21 (DE3)/E1-*GUS2*). (**C**) Results of HPLC determination (substrate: baicalin; product: baicalein). (**D**,**E**) UPLC-LTQ/Orbitrap MS primary (**D**) and secondary (**E**) results of GUS1 catalytic products. (**F**,**G**) The primary (**F**) and secondary (**G**) results of UPLC-LTQ/Orbitrap MS for GUS2 catalytic products.

The results for GUS3–GUS7 were omitted due to their negative outcomes in functional characterization experiments. The full-length ORF of *GUS1* and *GUS2* were successfully ligated into the prokaryotic expression vector. They resulted in amplifying nearly 2000 bp (Figure 3A). Using genetic engineering techniques, the recombinant expression plasmids pEASY-*GUS1* and pEASY-*GUS2* were successfully constructed and transformed into *E. coli* BL21 (DE3) cells. The empty vector E1 was transformed into *E. coli* BL21 (DE3) and served as a control. The SDS-PAGE results suggest that GUS1 and GUS2 were successfully expressed, and the molecular mass of recombinant enzymes were approximately 60 kDa and 55 kDa, respectively (Figure 3B).

In vitro catalytic products of the GUS1 and GUS2 proteins on the baicalin substrate were detected by HPLC. After incubation of the target proteins GUS1 and GUS2 with baicalin, chromatographic peaks consistent with the baicalein standard were detected, while no corresponding characteristic peaks were detected in the blank control sample (Figure 3C). This result preliminarily indicates that the target proteins GUS1 and GUS2 have catalytic activity for the conversion of baicalin to baicalein. The catalytic products of GUS1 and GUS2 were further qualitatively analyzed by UPLC-LTQ/Orbitrap MS (Figure 3D–G). The results further demonstrate that the excimer ion peak (*m*/*z*: 271.06 [M+H]^+^) of baicalein in the products catalyzed by both GUS1 (*m*/*z*: 270.97171 [M+H]^+^) and GUS2 (*m*/*z*: 271.06296 [M+H]^+^) recombinant proteins could be identified in the positive-ion mode. Secondary fragment ions were examined in more detail. Peaks in the GUS1 group included 252.98189 [M+H-H_2_O]^+^, 224.99953 [M+H-CO-H_2_O]^+^, and 168.93900 [M+H-C_8_H_6_]^+^, while the GUS2 group had 252.99078 [M+H-H_2_O]^+^, 224.86455 [M+H-CO-H_2_O]^+^, and 168.96663 [M+H-C_8_H_6_]^+^. These results further confirm that both the GUS1 and GUS2 proteins could catalyze the conversion of baicalin to baicalein.

### 2.5. Enzyme Kinetic Parameters of GUS1 and GUS2

**Figure 4:**
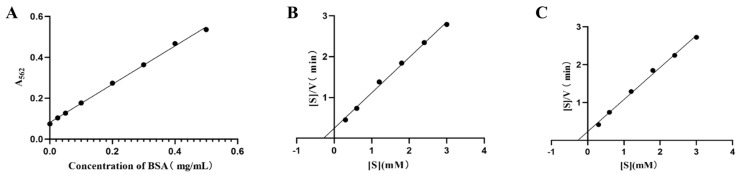
Results of enzyme kinetic parameters for GUS-catalyzed reactions. Standard curve for protein concentration determination (**A**), and kinetic analysis of GUS1 (**B**) and GUS2 (**C**).

The fitted linear regression equation for Y = 0.9362X + 0.08106 (R^2^ = 0.9976) was obtained by measuring the concentration of a protein standard solution using the BCA method (Figure 4A). According to the standard curve calculation results, the concentrations of the purified GUS1 and GUS2 proteins were 0.278 mg/mL and 0.384 mg/mL, respectively. The enzyme reaction rate (V) was calculated by determining the baicalein obtained from the GUS1 or GUS2 hydrolysis at different baicalin mass concentrations [S]. The enzymatic reaction rate is expressed in terms of the amount of product generated per minute. Scatter plots of [S]/V-[S] were plotted using the Hanes–Woolf method and were linearly fitted (Figure 4B,C). The fitting results of the parameters of enzyme reaction kinetics for GUS1 and GUS2 showed that the Km, Vmax, and Kcat of GUS1 were 0.2826 mg/mL, 1.1523 mg/(mL·min), and 41.4496 min^−1^, while for GUS2, they were 0.2732 mg/mL, 1.1797 mg/(mL·min), and 30.7214 min^−1^. These results show that GUS2 has better substrate affinity and enzymatic reaction rates than GUS1.

### 2.6. Expression and Catalytic Activity of GUS1 and GUS2 in Eukaryotic Systems

**Figure 5:**
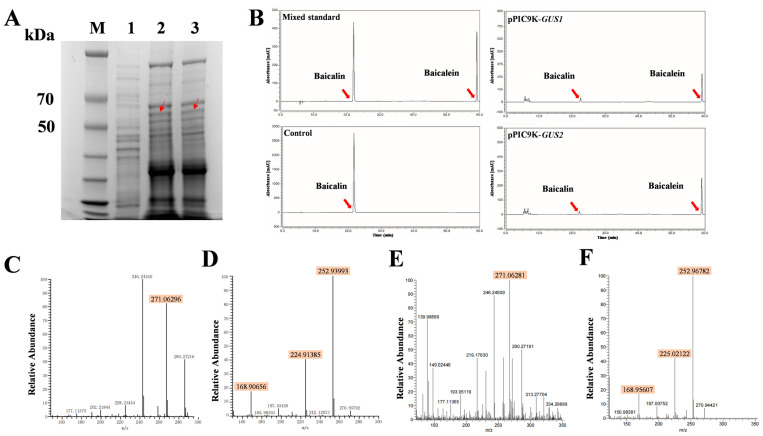
The eukaryotic expression experiment results of GUS1 and GUS2. (**A**) Expression of GUS1 and GUS2 in Pichia GS115 (M: Marker; 1: GS115/PIC9K; 2: GS115/PIC9K-*GUS1*; 3: GS115/PIC9K-*GUS2*). (**B**) The UV chromatograms of GUS1 and GUS2 catalytic results determined by HPLC (substrate: baicalin; product: baicalein). (**C**,**D**) UPLC-LTQ/Orbitrap MS primary (**C**) and secondary (**D**) results of GUS1 catalytic products. (**E**,**F**) The primary (**E**) and secondary (**F**) results of UPLC-LTQ/Orbitrap MS for GUS2 catalytic products.

The GUS1 and GUS2 target proteins can also be expressed in the Pichia pastoris GS115 eukaryotic system (Figure 5A). The catalytic activity of GUS1 and GUS2 was expressed and validated in eukaryotic systems, which was consistent with the catalytic results of prokaryotic expression systems. The HPLC results show that the baicalin substrate could be catalyzed by the GUS1 and GUS2 expressed in the eukaryotic system to convert into baicalein (Figure 5B). The product of baicalein was further characterized by UPLC-LTQ/Orbitrap MS (Figure 5C–F). In positive-ion mode, the primary fragment ion peaks of baicalein catalyzed by GUS1 and GUS2 were 271.06296 (Figure 5C) and 271.06281 (Figure 5E). Moreover, the three characteristic secondary fragment ion fronts were also detected and consistent with the prokaryotic experiment (Figure 5D,F). These results further confirm the stable catalytic activity of GUS1 and GUS2 in producing baicalein from baicalin.

### 2.7. Expression of GUS and its Correlation with Baicalin Accumulation

**Figure 6:**
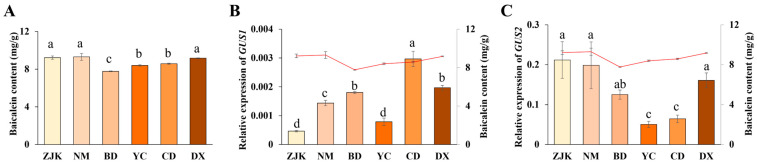
Results of baicalein content (**A**), *GUS1* expression (**B**), and *GUS2* expression (**C**) in *S. baicalensis* of different origins (*n* = 3). ZJK: Zhangjiakou; NM: Nei Mongolia; BD: Baoding, YC: Yuncheng; CD: Chengde; DX: Dingxi. Different letters indicate significant differences between the different groups (*p* < 0.05).

The determination results of baicalein content in six different sources of *S. baicalensis* seedlings show certain differences, among which the baicalein concentration in *S. baicalensis* from Nei Mongolia was the highest and in *S. baicalensis* from Baoding was the lowest (Figure 6A). And the Zhangjiakou group, Nei Mongolia group, and Dingxi group were classified as a high-content group based on the results of content determination, while the rest were classified as a low-content group. Simultaneously, the relative expression levels of *GUS1* and *GUS2* in these seedlings were evaluated by qRT-PCR (Figure 6B,C). The results show that *GUS1* had the highest expression level in Chengde *S. baicalensis* and the lowest expression level in Zhangjiakou *S. baicalensis*, while *GUS2* had the highest expression level in Zhangjiakou *S. baicalensis* and the lowest expression level in Yuncheng *S. baicalensis* (Figure 6B,C). In addition, the expression level of *GUS2* in *S. baicalensis* was generally higher than that of *GUS1*.

The correlation between the expression levels of the *GUS1* or *GUS2* gene and the content of baicalein in six origins of *S. baicalensis* was analyzed by SPSS 25 (Table S5). And the correlation analysis results show that the expression pattern of the *GUS1* gene was negatively correlated (−0.206) with the content of baicalein, while there was a significant positive correlation between the expression pattern of the *GUS2* gene and the content of baicalein (0.962) (*p* < 0.01). These results further imply that the accumulation of baicalein content in *S. baicalensis* was positively regulated by the expression of the *GUS2* gene.

To further analyze the differential activity of GUS in *S. baicalensis* from six origins, *GUS1* and *GUS2* were sequenced. The DNAMAN alignment analysis revealed that *GUS1* has two main variable sites in six different sources of *S. baicalensis*, while *GUS2* has six main variable sites (Table S6). The base changes of these variable sites in six different sources of *S. baicalensis* may further affect the catalytic activity of GUS proteins, leading to the formation of differences in baicalein content.

## 3. Discussion

Baicalein, a 5,6,7-trihydroxyflavone compound found in the root of *S. baicalensis*, has become a research hotspot because of its high bioavailability; excellent clinical safety; and various special pharmacological effects, such as antioxidant, anti-tumor, and antiviral effects [33,34]. Previously reported in vitro and in vivo experimental results show that baicalein was significantly more effective than baicalin in anti-cancer and anti-angiogenic effects [35]. In addition, animal experiments further confirmed that baicalein is the main form of substance absorbed by the gastrointestinal tract [36]. It is essential to conduct an in-depth study on the biosynthesis pathway of baicalein and its accumulation pattern in *S. baicalensis* in order to fully and effectively develop and apply it. Stress treatment is an effective strategy widely used to increase the content of secondary metabolites in medicinal plants, such as *S. baicalensis* [37], *Bupleurum chinense* [38], and licorice [39]. In this study, we first investigated the effect of 5% PEG6000 stress treatment on the content of baicalin (glycosides) and baicalein (aglycones) in *S. baicalensis* roots and found that their content was 1.69 and 1.20 times higher than that of the control group, respectively.

Glycoside hydrolases, as a superfamily, have numerous members in nature and are widely distributed and applied. *β*-glucuronidase (GUS), an important member of glycoside hydrolases, plays a crucial role in the conversion of glycosides and aglycones. The high-yield production of baicalein through hydrolysis using *β*-glucosidase has become an effective method, which was also used in clinical formulations [40]. At present, *β*-glucuronidases, used for preparing baicalein from baicalin, are mostly discovered in microorganisms, such as *Aspergillus niger* b.48 [41], *Lactobacillus rhamnosus* HP-B1083 [42], and *L. delbrueckii* Rh2 [43]. Although baicalin-*β*-D-glucuronidase has been isolated and identified from *S. viscidula* Bge [44] and *S. baicalensis* [12], its effect on baicalein accumulation has not been thoroughly explored. Based on a comparative transcriptomic analysis of *S. baicalensis*, seven candidate genes annotated as *β*-glucuronidase were identified from differentially expressed genes in this study. And the conservative domain prediction results of the seven candidate genes show that they all belong to the glycosyl hydrolase 79 superfamily. Seven candidate GUSs were induced to express in prokaryotic systems, and GUS1 and GUS2 were identified to have catalytic activity for the production of baicalein from the substrate baicalin derived from in vitro enzymatic reactions (Figure 3). And the enzymatic kinetic parameters indicate that GUS2 has better substrate affinity and catalytic rates compared to GUS1 (Figure 4). The conversion activity of GUS1 and GUS2 towards baicalin was also further confirmed in the Pichia GS115 system (Figure 5). Genetic genes will experience various degrees of variation over the long-term development of plants due to natural mutations, leading to gene polymorphisms, of which the polymorphism of functional genes has a substantial effect on the synthesis of regulatory products [45]. This study further analyzed the polymorphism of the *GUS1* and *GUS2* gene sequences in six different sources of *S. baicalensis.* The *GUS* of *S. baicalensis* has a wide range of genetic polymorphisms, with two variable sites in *GUS1* between high- and low-baicalin-content groups and six major variable sites in *GUS2* between high- and low-baicalin-content groups. It is speculated that these sites may be related to the levels of flavonoid compounds. This study can guide further polymorphism analyses of functional genes in *S. baicalensis*, laying the foundation for elucidating the relationship between secondary metabolism and functional genes.

The synthesis and accumulation of secondary metabolites in plants are controlled by key enzymes in the biosynthetic pathway, and the expression level of enzyme genes is closely related to the accumulation of active ingredients [46,47]. Although previous studies have found that endogenous *sbGUS* in *S. baicalensis* has a high expression level in roots and have proposed that this gene may be involved in the biosynthesis of baicalein, further verification has not been obtained [11]. This study detected the content of baicalein and the expression levels of the *GUS1* and *GUS2* genes in *S. baicalensis* seedlings treated with 5% PEG6000 from six different sources. An additional correlation analysis confirmed a significant positive correlation between the expression level of the *GUS2* gene and the content of baicalein in *S. baicalensis*. This study indicates that the *GUS2* gene may play a more important role in regulating the content of baicalein in *S. baicalensis* compared to the *GUS1* gene.

This study serves as an important reference for the molecular mechanism of endogenous *GUS* genes in regulating baicalein accumulation in *S. baicalensis*. The endogenous GUS1 and GUS2 in *S. baicalensis* have the activity of catalyzing the production of baicalein from baicalin, and the expression level of *GUS2* was significantly positively correlated with the accumulation of baicalein. In summary, the results of this study provide new insights into the accumulation of baicalein in *S. baicalensis*.

## 4. Materials and Methods

### 4.1. Plant Material and Chemicals

*S. baicalensis* seeds from six different geographic locations were collected as the experimental material, including Nei Mongolia, Zhangjiakou, Baoding, Yuncheng, Chengdu, and Dingxi. All seeds were dried and stored in a refrigerator for this study. The full and healthy seeds of *S. baicalensis* were selected and planted in pots for further experiments. The samples were identified as *S. baicalensis* by Prof. Chunsheng Liu, School of Traditional Chinese Medicine, Beijing University of Traditional Chinese Medicine.

The TRNzol Universal Total RNA Extraction Reagent (DP424) was produced by TianGen (Beijing, China). The pEASY^®^-Blunt Zero Cloning Kit (CB501-01), pEASY^®^-Blunt E1 Expression Kit (CE111-01), and BL21 (DE3) Chemically Competent Cell (CD601-02) were purchased from TransGen Biotech (Beijing, China). The His-tag Protein Purification Kit (P2226) and BCA Protein Assay Kit (P0012S) were bought from Beyotime Biotechnology (Shanghai, China). The Evo M-MLV Mix Kit with gDNA Clean for the qPCR kit (AG11705) was produced by AGBio (Changsha, China). The ChamQ Universal SYBR qPCR Master Mix (Q711-02/03) and 2 × Phanta Flash Master Mix (Dye Plus) (P520) were obtained from Vazyme (Nanjing, China). The reagents used for the experiments were baicalin (HPLC; CAS: 491-67-8) and baicalein (HPLC; CAS: 491-67-8), purchased from Yuanye (Shanghai, China). MS grade formic acid (CAS: 6449-79-2) and acetonitrile (CAS: 75-05-8) were purchased from Thermo Fisher Scientific (Waltham, MA, USA). All other chemicals typically were of analytical grade and bought locally.

### 4.2. Stress Treatment

The collected seeds of *S. baicalensis* were washed several times with water and then sown equally in pots (nutrient soil:vermiculite:perlite = 2:1:1). The seedlings were incubated at room temperature, 50% humidity, 16 h of light, and 8 h of darkness. After four weeks of growth, the seedlings were treated with H_2_O and 5% PEG6000 for five days, with three replicates for each treatment. The content of baicalin and baicalein in the *S. baicalensis* roots was determined by HPLC. Seedlings were promptly frozen in liquid nitrogen after the treatment and stored at −80 °C.

The Nei Mongolia *S. baicalensis* seedlings treated with 5% PEG6000 were used for further analysis to identify GUS genes that may be involved in baicalein synthesis and to perform a functional characterization. On this basis, the *S. baicalensis* seedlings treated with 5% PEG6000 from Nei Mongolia, Zhangjiakou, Baoding, Yuncheng, Chengde, and Dingxi were used to analyze the relationship between *GUS* expression and baicalein content and differences in the catalytic efficiency of *GUS*-encoded enzymes from various sources.

### 4.3. Transcriptome Sequencing and Analysis

The total RNA was extracted from the roots, stems, and leaves of the *S. baicalensis* seedlings treated with H_2_O and 5% PEG6000 using the Trizol method. The mRNA was subsequently isolated from the total RNA, reverse transcribed into cDNA, and ligated to adaptors to construct sequencing libraries. The quality of the libraries was assessed using a Nanodrop 2000 (Thermo Scientific, Waltham, MA, USA), Qubit 2.0 Fluorometer (Thermo Scientific, Waltham, MA, USA), and Agilent 2100 Bioanalyzer (Agilent, Santa Clara, CA, USA). After the libraries passed the inspection, high-throughput sequencing was performed with Illumina xplus sequencing platform. The data of the sequenced fragments, measured using the high-throughput sequencing instrument, were converted into reads after base recognition through CASAVA. Low-quality reads were filtered out, and the sequencing error rate and GC content distribution were evaluated. Unigenes, obtained by splicing, were annotated by gene function, and the differential expression was analyzed.

### 4.4. RNA Extraction, cDNA Synthesis, and GUS Cloning

cDNA was obtained by reverse transcription based on the instructions of the Evo M-MLV Mix Kit with gDNA Clean for the qPCR kit. The full-length sequences of candidate *GUSs* were obtained from the transcriptome data of *S. baicalensis*. PCR amplification was performed by designing specific primers (Table S3), using *S. baicalensis* cDNA as a template. The amplification system was as follows: 2 × Phanta Flash Master Mix (Dye Plus) 15 μL of the primers F and R, 1 μL each; 1 μL of the template; and the rest was made up of sterile double-distilled water, 30 μL. The reaction conditions were as follows: pre-denaturation at 98 °C for 30 s, denaturation at 98 °C for 10 s, annealing at 58 °C for 5 s, extension at 72 °C for 9 s for 35 cycles, followed by extension at 72 °C for 1 min. PCR amplification products were detected by 1% gel electrophoresis and then stored in a refrigerator at −20 °C.

### 4.5. Sequence Alignment and Bioinformatics Analysis

The complete coding region sequences of the candidate *GUS* genes were obtained by splicing the two-way sequencing peak map and removing the weak or overlapped peak areas at both ends by applying the Contig Express (June 20, 2000) software. And the amino acid sequences of the candidate *GUS* genes were translated using the EditSeq (v. 7.1.0) software. The structural domains of the candidate proteins were predicted by the NCBI Conserved Domain Search tool. The subcellular localization of the proteins was predicted using the WoLF PSORT subcellular localization prediction online (https://www.genscript.com/psort.html, accessed on 1 January 2025). ExPASy-ProtParam online (https://web.expasy.org/protparam/, accessed on 1 January 2025) was used to predict the physicochemical properties of the candidate proteins. *GUS* sequences from bacteria, fungi, animals, and plants were downloaded from NCBI for a phylogenetic analysis to examine the evolutionary relationship of the candidate *GUSs* among various species. A phylogenetic tree of *GUSs* was generated using MEGA 10, based on a neighbor-joining (NJ) method with 1000 replications of bootstrapping.

### 4.6. Construction of Prokaryotic Expression Vectors and In Vitro Expression of GUSs

The PCR amplification products of the candidate *GUS* genes were purified by gel recovery and then mixed with the expression vector pEASY^®^-Blunt E1 at 25 °C for 30 min to obtain recombinant plasmid E1-GUSs. The recombinant plasmid E1-*GUSs* were transformed into *E. coli* Trans1-T1 receptor cells, and a single clone was selected for sequencing. The positive clones of the plasmid E1-GUSs were extracted from the expanded culture of the bacterial broth and transformed into BL21 (DE3) receptor cells. Empty vector pEASY^®^-Blunt E1 was used as a control, and positive clones with recombinant plasmids were screened by PCR, amplified using primers T7 and T7T, and were named as BL21 (DE3)/E1-*GUS1* and BL21 (DE3)/E1-*GUS2*. The BL21 (DE3)/E1-*GUS1* and BL21 (DE3)/E1-*GUS2* transformants were inoculated in an LB medium supplemented with 100 mg/mL of ampicillin and incubated at 37 °C. When the OD_600_ of the culture medium reached 0.6~0.8, IPTG, with a final concentration of 0.2 mmol/L, was added to the culture medium and then incubated for 18 h at 16 °C. The culture liquid was centrifuged at 4 °C and 8000 rpm for 20 min, and the supernatant was discarded. The collected cell pellet was re-dissolved in 5 mL of a phosphate buffer solution. The resuspended cell solution was sonicated in an ice water bath for 30 min, and the solution was centrifuged at 4 °C and 12,000 rpm for 20 min. The supernatant was collected for an SDS-PAGE analysis, and the successfully expressed protein solution was used for subsequent in vitro catalytic activity testing and purification.

### 4.7. In Vitro Catalytic Activity of Candidate GUSs on Baicalin

The catalytic activity of GUS1 and GUS2 towards the baicalin substrate was detected through an in vitro enzymatic reaction. A total of 20 mL of baicalin (1 mM), 10 mL of a GUS enzyme solution, and 70 mL of PBS (pH 9) were mixed to form a reaction system and incubated at 35 °C for one hour. After the reaction, an equal volume of methanol was directly added to stop the reaction, and the control group used an equal volume of a PBS solution instead of a GUS enzyme solution. A mixed standard solution of 1 mM of baicalin and 1 mM of baicalein was prepared with methanol. HPLC was used for the identification of enzymatic reaction products in vitro. The determination conditions for HPLC were as follows: the mobile phase was an acetonitrile (A) −0.1% formic acid aqueous solution (B). The gradient elution was used under the following conditions: 0~4 min, 10~20% A; 4~12 min, 20~22% A; 12~22 min, 22~24% A; 22~49 min, 24~28% A; 49~52 min, 28~35% A; 52~60 min, 35~45% A; 60~64 min, 45~55% A; 64~70 min, 55~55% A. 45~55% A; 64~70 min, 55~10% A; and 70~85 min, 10% A. The volume flow rate was 0.8 mL/min, the detection wavelength was 274 nm, the injection volume was 10 μL, and the column temperature was 30 °C.

Qualitative detection was performed using UPLC-LTQ/Orbitrap MS (Thermo Scientific, Karlsruhe, Germany). The analytical conditions were as follows: the Waters ACQUITY UPLC T3 column (2.1 mm × 100 mm, 1.7 m, Waters, Milford, MA, USA) was used; the mobile phase was a 0.1% formic acid−aqueous solution, (A) acetonitrile−(B) gradient elution (0 min, 99% A; 2 min, 99% A; 4 min, 83% A; 14 min, 81% A. 15 min, 5% A; and 17 min, 5% A); the flow rate was 0.30 mL/min; the column temperature was 40 °C; and the injection volume was 1 μL. The LTQ Oribitrap XL (Thermo Scientific, Germany) linear ion trap series electrostatic field orbital trap mass spectrometer, equipped with a hot spray ion source (HESI) and Xcalibur 2.1 ChemStation (v.4.1.31.9), operates in negative-ion detection mode. The ion source temperature was 350 °C, the ionization source voltage was 4 KV, the capillary voltage was 35 V, the tube lens voltage was 110 V, and both the sheath gas and auxiliary gas were high-purity nitrogen gas (purity of >99%). The sheath gas flow rate was 40 arb, the auxiliary gas flow rate was 20 arb, and the data were collected using Fourier transform high-resolution full-scan-mode data dependent on ddMS^2^ and ddMS^3^.

### 4.8. Purification of Recombinant Proteins and Catalytic Parameters

The GUS1 and GUS2 protein solutions were purified by the His-tag Protein Purification Kit. The protein concentration was determined following the directions of the BCA Protein Assay Kit after the purified protein solution had been concentrated using Amicon^®^ Ultra ultrafiltration tubes (15 mL; 30 k) (Merck, Darmstadt, Germany).

An in vitro enzymatic reaction was carried out using baicalin at different concentrations as the substrate. The following were mixed and incubated at 45 °C for 40 min: a total of 30 μL of the substrate (0.3–3 mM), 10 μL of the enzyme solution, and 60 mL of a PBS (pH = 9). The reaction was terminated by adding an equal volume of methanol. The HPLC method mentioned in Section 4.7 was used to calculate catalytic parameters. Kinetic parameters were calculated and plotted using GraphPad Prism 8.3.0.

### 4.9. Analysis of Enzyme Activity by Eukaryotic Expression System

The catalytic activity of GUS1 and GUS2 towards baicalin was further validated through yeast eukaryotic expression. The recombinant yeast expression vectors pPIC9K-GUS1 and pPIC9K-GUS2 were constructed by PCR amplification, with the pEASY-*GUS1* and pEASY-*GUS2* vectors as templates. G1F, G1R, G2F, and G2R homology arm primers were created using the properties of the sequence (Table S3). The pPIC9K vector and *GUS1* and *GUS2* PCR products were digested by *EcoR*I and *Not*I, and then, the amplified products were ligated with pPIC9K. The linearized recombinant plasmids pPIC9K-*GUS1* and pPIC9K-*GUS2*, digested with Sal I, were transformed into a competent cell of pichia GS115 by electroporation. The transformed pichia cells were sequentially screened by His^+^, G418, and Mut^+^, and the positive transformants were named as GS115/pPIC9K-*GUS1* and GS115/pPIC9K-*GUS2*. And the control GS115/pPIC9K was obtained by transferring the empty vector pPIC9K into GS115. GS115/PIC9K-*GUS1* and GS115/PIC9K-*GUS2* were sequentially inoculated in a YPD liquid medium, a BGMY medium, and a BMMY medium at a ratio of 1:100 and incubated for 24 h in a shaker at 30 °C and 180 rpm. Then, 1% (*v*/*v*) methanol was added to the BMMY liquid medium and induced for 7 days in a shaker at 30 °C and 250 rpm. And methanol was added every 24 h to maintain a concentration of 1%. The induced culture solution was centrifuged and resuspended in 6 mL of a PBS solution and then sonicated for 40 min. The supernatant of the target protein GUS1 and GUS2 solutions were collected by centrifugation at 4 °C for 15 min and used for subsequent protein detection and catalytic reaction of baicalin. The catalytic reaction system and method for determining the baicalein produced from the reaction were the same as described in Section 2.7.

### 4.10. Correlation Analysis of GUS Expression and Baicalin Accumulation

One-month-old seedlings of *S. baicalensis* from 6 origins (Zhangjiakou, NeiMongolia, Baoding, Yuncheng, Chengde, and Dingxi) were treated with 5% PEG6000, and the total RNA of the roots was extracted. The cDNA was obtained from the extracted RNA by reverse transcription, according to the instructions of the Evo M-MLV Mix Kit with gDNA Clean for the qPCR kit. The primers (Q1F, Q1R, Q2F, and Q2R) of *GUS1* and *GUS2* were designed for qRT-PCR analyses. qRT-PCR assays were carried out following the ChamQ Universal SYBR qPCR Master Mix using an SYBR Green-based PCR assay. The expression levels of *GUS1* and *GUS2* were determined using CT values and calculated using the 2^−ΔΔCt^ method. Three biological and technical replicates were carried out for each analysis. Meanwhile, the content of baicalein in the roots of *S. baicalensis* from 6 different sources was treated with 5% PEG6000 by the HPLC method mentioned in Section 4.7. Then, the correlation between the expression of *GUS1* and *GUS2* and the content of baicalein was analyzed separately by the Pearson method using SPSS 25.

### 4.11. Statistical Analysis

GraphPad Prism 8.3.0 and Adobe Illustrator 2020 were used for data computation and image drawing. The statistical software IBM SPSS^®^ Statistics 25 (v.25.0.0) was used for data analysis. After conducting homogeneity of variance tests on the experimental data, variance analysis was used to analyze the significance between different treatments, and the LSD test was used for multiple comparisons.

## 5. Conclusions

In summary, the use of 5% PEG6000 treatment in this study significantly (*p* < 0.01) increased the content of baicalin and baicalein in *S. baicalensis* by 68.72% and 20.44%, respectively. Seven candidate *GUS* DEGs, belonging to GH79 family, were further identified through comparative transcriptomics. Furthermore, it was confirmed, through prokaryotic and eukaryotic expression systems, that endogenous GUS1 and GUS2 in *S. baicalensis* have the function of catalyzing the hydrolysis of baicalin to baicalein, and GUS2 has better substrate affinity and enzymatic reaction rates than GUS1. The correlation analysis results confirm a significant positive correlation (0.962; *p* < 0.01) between the expression level of the endogenous *GUS2* gene and the content of baicalein in the six different sources of *S. baicalensis.* In addition, this study provides a new insight into the accumulation of baicalein-active ingredients in *S. baicalensis* and also provides key enzyme resources for the effective conversion or synthesis of baicalein. However, further in-depth research is needed to investigate the effects of variable-site base changes of *GUS* genes in *S. baicalensis* from different sources on its catalytic activity.

## Data Availability

The raw transcriptome data of the *S. baicalensis* generated in this study can be obtained from NCBI (search: PRJNA910597-NLM). The original contributions presented in this study are included in the article/Supplementary Materials. Further inquiries can be directed at the corresponding author.

## Supplementary Materials

The following supporting information can be downloaded at: https://www.mdpi.com/article/10.3390/ijms26051793/s1.
