# Supplementary material for: Functional Characterization of β-Glucuronidase Genes Involved in Baicalein Biosynthesis from Scutellaria baicalensis Based on Transcriptome Analysis
Source: Int J Mol Sci. 2025 Feb 20;26(5):1793. doi: 10.3390/ijms26051793 (PMC11898752; doi:10.3390/ijms26051793)
Supplement: Supplementary file 1 [file ijms-26-01793-s001.zip › ijms-3434099-supplementary.pdf]

# Functional Characterization of *β*-Glucuronidase Genes Involved in Baicalein Biosynthesis from *Scutellaria baicalensis* Based on Transcriptome Analysis

**Xin Zuo †, Ping Li †, Guangxi Ren, Zhenfang Bai, Dan Jiang * and Chunsheng Liu ***

School of Chinese Materia Medica, Beijing University of Chinese Medicine, Beijing 102488, China
* Correspondence: 201801010@bucm.edu.cn (D.J.); liucs@bucm.edu.cn (C.L.)
† These authors contributed equally to this work.

## Supplementary materials Legends:

**Figure S1. Results of functional gene annotation.**

**Figure S2. Gel electrophoresis of the seven cloned GUS genes.**

**Figure S3. Structural prediction of GUSs proteins.**

**Table S1. Quality statistics of transcripts and unigenes data.**

**Table S2. Distribution of transcriptome data splicing length.**

**Table S3. Information on the primers used in this study.**

**Table S4. Results of the bioinformatics analysis of GUS1 to GUS7 proteins.**

**Table S5. The correlation between baicalein content and *GUS1* and *GUS2* gene expression levels in six different sources of *S. baicalensis* roots.**

**Table S6. Major variable sites baicalin of *GUS1* and *GUS2* in low/high content of baicalein in *S.baicalensis*.**

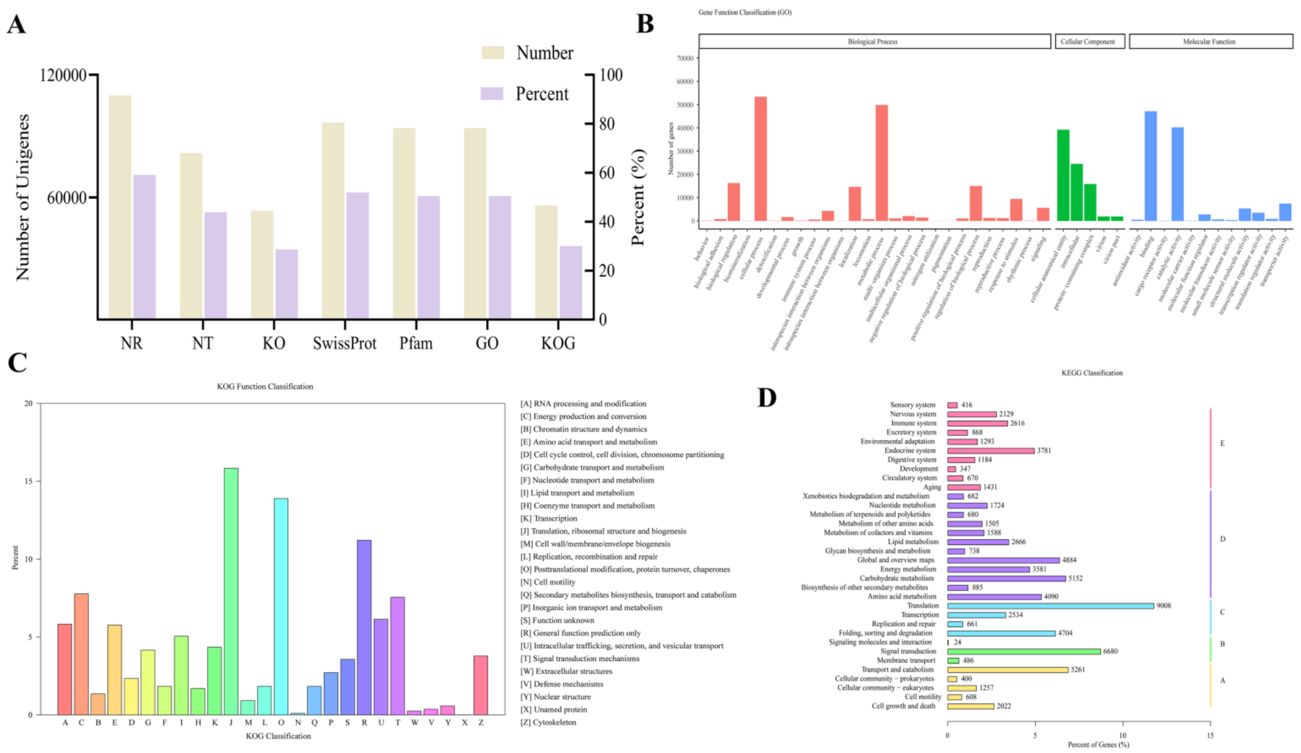


**Figure S1. Results of functional gene annotation. (A)** Gene annotation success rate statistics. Statistical chart of annotation classification of GO **(B)**, KOG **(C)**, and KEGG **(D)**.

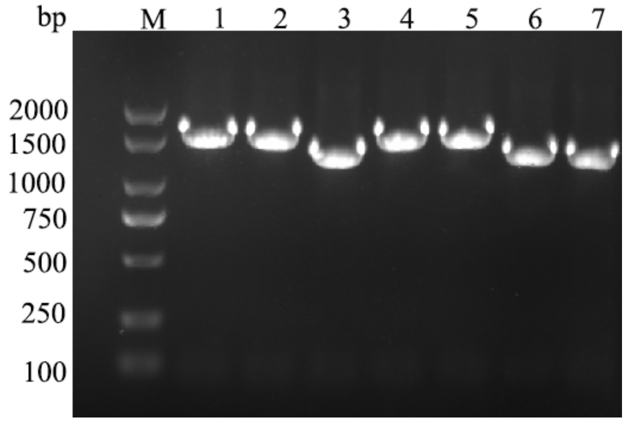


**Figure S2. Gel electrophoresis of the seven cloned GUS genes** (M: marker; 1: *GUS1*; 2: *GUS2*; 3: *GUS3*;4: *GUS4*; 5: *GUS5*; 6: *GUS6* ;7: *GUS7*)**.**

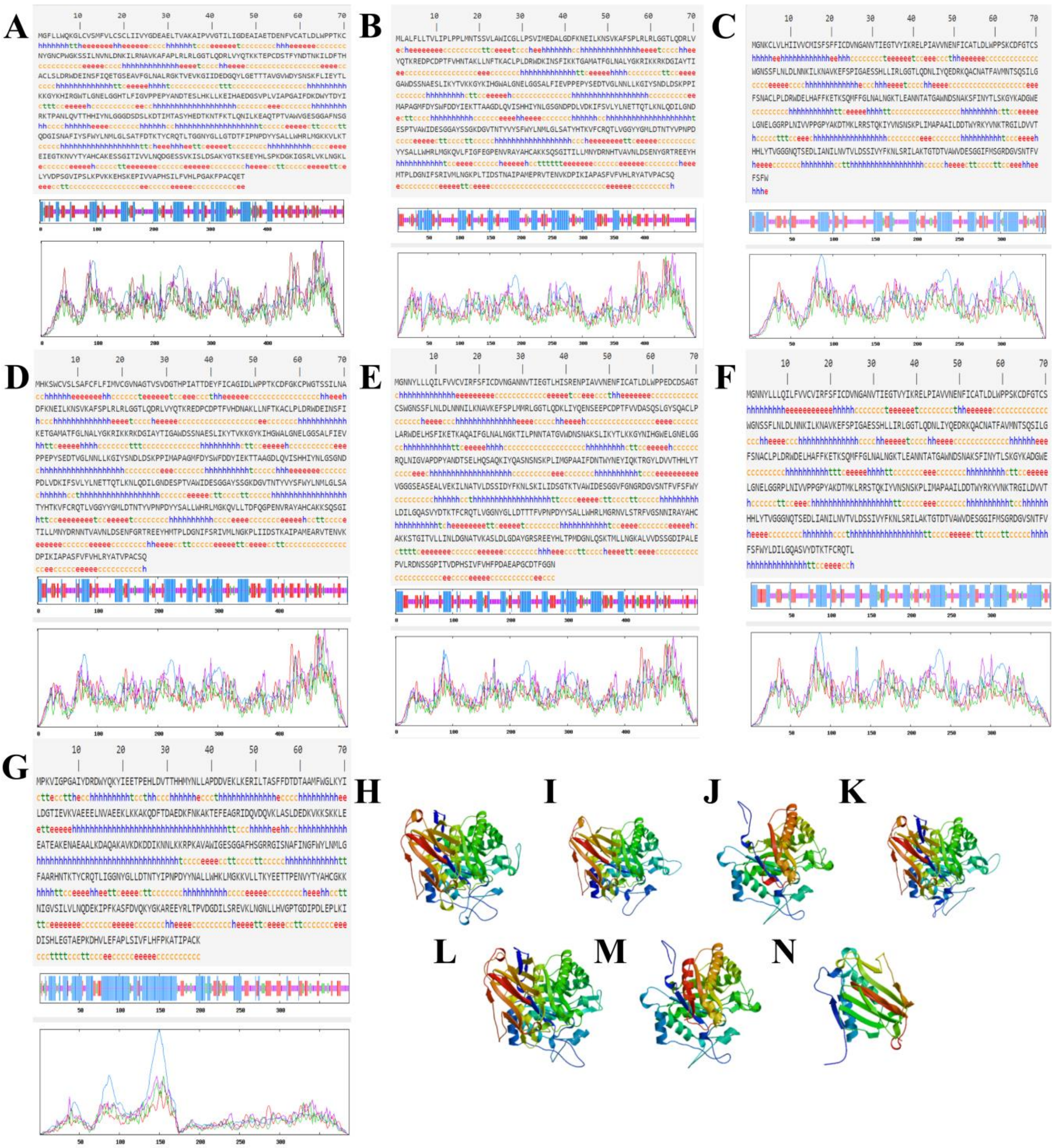


**Figure S3. Structural prediction of GUSs proteins.** Secondary structure prediction of GUS1 **(A)**, GUS2 **(B)**, GUS3 **(C)**, GUS4 **(D)**, GUS5 **(E)**, GUS6 **(F)**, and GUS7 **(G)**. 3D structure prediction of GUS1 **(H)**, GUS2 **(I)**, GUS3 **(J)**, GUS4 **(K)**, GUS5 **(L)**, GUS6 **(M)**, and GUS7 **(N)**.

**Table S1. Quality statistics of transcripts and unigenes data.**

| No. | Describe | raw_reads | clean_reads | error_rate(%) | Q20(%) | Q30(%) | GC_pct(%) |
|---|---|---|---|---|---|---|---|
| 1 | Leaves of 5%PEG6000 treated | 38293964 | 36975669 | 0.02 | 98.33 | 94.89 | 45.34 |
| 2 | Roots of 5%PEG6000 treated | 39630781 | 37246057 | 0.02 | 98.18 | 94.63 | 47.06 |
| 3 | Stems of 5%PEG6000 treated | 39910008 | 38005211 | 0.03 | 97.89 | 93.85 | 45.43 |
| 4 | Leaves of 0%PEG6000 treated | 41654964 | 40263368 | 0.02 | 98.02 | 94.2 | 46.27 |
| 5 | Roots of 0%PEG6000 treated | 39138890 | 37175242 | 0.02 | 98.07 | 94.45 | 47.99 |
| 6 | Stems of 0%PEG6000 treated | 41625643 | 39967107 | 0.02 | 98.31 | 94.97 | 43.96 |

**Table S2. Distribution of transcriptome data splicing length.**

| | Total | Min_length(bp) | Mean_length(bp) | Max_length(bp) | N50 | N90 |
|---|---|---|---|---|---|---|
| Transcripts | 312177 | 301 | 1034 | 14401 | 1712 | 404 |
| Unigenes | 186042 | 301 | 745 | 14401 | 926 | 353 |

**Table S3. Information on the primers used in this study.**

| Name | Primer sequences（5’-3’） |
|---|---|
| GUS1F | ATGGGTTTTCTGCTTTGGCAAAAGG |
| GUS1R | TCAGGTTTCTTGACATGCAGGGAAC |
| GUS2F | ATGCTGGCACTGTTTCTGTTGACGG |
| GUS2R | CTATTGAGAGCATGCAGGGACAGTA |
| GUS3F | AAGATGGGTAATAAATGTCTGG |
| GUS3R | TGTGATCGGTTAGTTGAAGC |
| GUS4F | ATGCATAAGAGTTGGTGTGTTTC |
| GUS4R | CATAGTAGAGCAAAGTAGTGAAGG |
| GUS5F | TAGCTGAAGTTGTAGTAAAGATGGGC |
| GUS5R | TATCAGTTCCCTCCAAATGTATCAC |
| GUS6F | ATGGGCAATAATTATCTGCTTTTGCAG |
| GUS6R | CTAATGTCTGTCTACAGAATGTCTTCGTGT |
| GUS7F | GAGTGTTTGTCCTTAGAGCGATG |
| GUS7R | GCTATTCAAACTCCAGGCTGATTAC |
| G1F | CTGAAGCTTACGTAGATGGGTTTTCTGCTTTGGCAAA |
| G1R | GGCGAATTAATTCGCTCAGGTTTCTTGACATGCAGGG |
| G2F | CTGAAGCTTACGTAGATGCTGGCACTGTTTCTGTTGA |
| G2R | GGCGAATTAATTCGCTTATTGAGAGCATGCAGGGACA |
| Q1F | GGGTTGGTGAATCTGGAGGA |
| Q1R | GCCACCAGTCAATGTCTGTC |
| Q2F | CCCTGTGATTCCACCTTCTAC |
| Q2R | CGGCTTCAGACCCAGTTT |

**Table S4. Results of the bioinformatics analysis of GUS1 to GUS7 proteins.**

| Genes | ORF (bp) | Amino acid | MW (kDa) | pI | II | GRAVY | Alpha helix (%) | Hydropathicity | Stability | Subcellular localization |
|---|---|---|---|---|---|---|---|---|---|---|
| *GUS1* | 1614 | 537 | 59.30 | 5.90 | 35.35 | -0.187 | 28.86 | Hydropathic | Stability | cytoplasm |
| *GUS2* | 1458 | 485 | 54.03 | 6.28 | 36.66 | -0.142 | 29.28 | Hydropathic | Stability | cytoplasm |
| *GUS3* | 1065 | 354 | 39.15 | 6.94 | 39.01 | - 0.027 | 35.88 | Hydropathic | Stability | cytoplasm |
| *GUS4* | 1548 | 515 | 57.37 | 5.91 | 35.28 | -0.179 | 27.77 | Hydropathic | Stability | cytoplasm |
| *GUS5* | 1584 | 527 | 57.68 | 5.07 | 34.61 | -0.135 | 27.89 | Hydropathic | Stability | cytoplasm |
| *GUS6* | 1126 | 375 | 41.66 | 6.89 | 36.99 | -0.045 | 38.67 | Hydropathic | Stability | cytoplasm |
| *GUS7* | 1164 | 397 | 43.73 | 6.05 | 26.33 | -0.525 | 41.09 | Hydropathic | Stability | cytoplasm |

**Table S5. The correlation between baicalein content and *GUS1* and *GUS2* gene expression levels in six different sources of *S. baicalensis* roots.**

| | ***GUS1* relative expression** | ***GUS2*relative expression** |
|---|---|---|
| **Baicalein content** | -0.206 | 0.962** |
| ***p*_value** | 0.275 | 0.002 |

**Table S6 Major variable sites baicalin of *GUS1* and *GUS2* in low/high content of baicalein in *S.baicalensis*.**

| Gene | bp | Low content | High content |
|---|---|---|---|
| GUS1 | 753 | G | A |
| | 808 | G | A |
| GUS2 | 72 | G | A |
| | 101 | A | T |
| | 107 | G | A |
| | 729 | G | C |
| | 766 | G | A |
| | 777 | A | T |
